# Overexpression of c-Jun contributes to sorafenib resistance in human hepatoma cell lines

**DOI:** 10.1371/journal.pone.0174153

**Published:** 2017-03-21

**Authors:** Yuki Haga, Tatsuo Kanda, Masato Nakamura, Shingo Nakamoto, Reina Sasaki, Koji Takahashi, Shuang Wu, Osamu Yokosuka

**Affiliations:** 1 Department of Gastroenterology and Nephrology, Chiba University, Graduate School of Medicine, Chiba, Japan; 2 Department of Molecular Virology, Chiba University, Graduate School of Medicine, Chiba, Japan; Hunter College, UNITED STATES

## Abstract

**Background:**

Despite recent advances in treatment strategies, it is still difficult to cure patients with hepatocellular carcinoma (HCC). Sorafenib is the only approved multiple kinase inhibitor for systemic chemotherapy in patients with advanced HCC. The majority of advanced HCC patients are resistant to sorafenib. The mechanisms of sorafenib resistance are still unknown.

**Methods:**

The expression of molecules involved in the mitogen-activated protein kinase (MAPK) signaling pathway in human hepatoma cell lines was examined in the presence or absence of sorafenib. Apoptosis of human hepatoma cells treated with sorafenib was investigated, and the expression of Jun proto-oncogene (c-Jun) was measured.

**Results:**

The expression and phosphorylation of c-Jun were enhanced in human hepatoma cell lines after treatment with sorafenib. Inhibiting c-Jun enhanced sorafenib-induced apoptosis. The overexpression of c-Jun impaired sorafenib-induced apoptosis. The expression of osteopontin, one of the established AP-1 target genes, was enhanced after treatment with sorafenib in human hepatoma cell lines.

**Conclusions:**

The protein c-Jun plays a role in sorafenib resistance in human hepatoma cell lines. The modulation and phosphorylation of c-Jun could be a new therapeutic option for enhancing responsiveness to sorafenib. Modulating c-Jun may be useful for certain HCC patients with sorafenib resistance.

## Introduction

The estimated number of new cases of liver cancer in 2012 was 782,000 worldwide, including 554,000 and 228,000 cases in men and women, respectively [1]. The estimated number of cancer deaths from liver cancer in 2012 was 745,000 worldwide, including 521,000 and 224,000 deaths in men and women, respectively [1]. The very small difference between the numbers of new cases and deaths from liver cancer indicates a poor prognosis. Among liver cancers, hepatocellular carcinoma (HCC) is the most common primary liver cancer. Decompensation of liver function and the development of HCC are dreaded complications of advanced liver diseases. The annual incidence of HCC in the adult Taiwanese population remains high despite the fact that here has been a more than 50% drop in HCC incidence following national hepatitis B virus (HBV) vaccination programs in Taiwan [2]. A large population with chronic HBV infection remains at risk of developing cirrhosis and HCC if left untreated [2]. Recent progress in treatments for the hepatitis C virus (HCV) has been shown to significantly alter the natural progression to HCC in countries with HCV as a major contributor to HCC [3]. However, a large population with chronic HCV infection is still at risk of developing cirrhosis and HCC if left untreated [3]. Despite the progress in imaging modalities, it is still difficult to detect the early stages of HCC [4]. Other than a liver transplantation, it is difficult to cure patients with HCC because many of the patients have liver cirrhosis [4].

Sorafenib is the only approved multiple kinase inhibitor for the systemic chemotherapeutic reagents for compensated cirrhotic patients with unresectable or metastatic HCC, although the complete response rate to sorafenib in HCC is relatively low (0.7%-3%) [5]. Molecular targets of sorafenib are tyrosine kinases of the vascular endothelial growth factor receptor (VEGFR) and platelet-derived growth factor receptor (PDGFR) [6]. Sorafenib also exerts its effects by targeting mitogen-activated protein kinase (MAPK) kinase kinase (Raf)/MAPK kinase (MEK)/MAPK [originally called extracellular signaling-related kinase (ERK)] signaling at the level of Raf kinase [6,7]. The success of anticancer treatment with sorafenib would depend on having a better understanding of its acquired resistance mechanism in HCC [7].

Stress-activated protein kinases (SAPKs)/Jun proto-oncogene (c-Jun) N-terminal kinases (JNKs) are members of the MAPK family that are activated by cellular environmental stresses, inflammatory cytokines and growth factors [8, 9]. JNK1 binds to the c-Jun transactivation domain and phosphorylates c-Jun, and JNK1 activation plays a role in tumor promotion [8]. The JNK signaling pathway plays an important role in cellular apoptosis [10] and in a cisplatin (CDDP) resistance mechanism in cancer cells [9]. A previous study [10] showed that the transcription factor c-Jun/AP-1 promoted HBV-related liver tumorigenesis in mice.

In the present study, we demonstrated that c-Jun was elevated in human hepatoma cells treated with sorafenib. We report that the expression and phosphorylation of c-Jun conferred sorafenib resistance in human hepatoma cells. These mechanisms might play an important role in the chemoresistance of HCC patients treated with sorafenib.

## Materials and methods

### Cell culture

Human hepatoma cell lines Huh7, Huh6, PLC/PRF/5, Hep3B, HepG2 and HepG2.2.15 were grown in Roswell Park Memorial Institute medium (RPMI1640) (Sigma-Aldrich, St. Louis, MO, USA) supplemented with 10% fetal bovine serum (FBS), 200 U/mL of penicillin, and 200 μg/mL of streptomycin at 5% CO_2_ and 37°C. Huh7, HepG2 and HepG2.2.15 were previously reported [11, 12]. Huh6 and PLC/PRF/5 were purchased from the National Institutes of Biomedical Innovation, Health and Nutrition JCRB Cell Bank (Ibaraki, Osaka, Japan). Hep3B cells were obtained from American Type Culture Collection (ATCC) (Manassas, VA, USA).

### Reagents

Sorafenib and JNK inhibitor SP600125 were purchased from Cayman Chemical (Ann Arbor, MI, USA) and AdooQ BioScience (Irvine, CA, USA), respectively.

### Western blotting

Cells were collected in 1% sodium dodecyl sulfate (SDS) buffer. After sonication, proteins were subjected to electrophoresis on a 5–20% SDS-polyacrylamide gel and transferred onto a polyvinylidene difluoride membrane (ATTO, Tokyo, Japan) followed by overnight blocking with 5% skim milk in phosphate-buffered saline with Tween 20 (Bio-Rad, Hercules, CA, USA). The membrane was probed with antibodies specific to phosphorylation of c-Jun [p-c-Jun (Ser63)], c-Jun (Cell Signaling, Boston, MA, USA), osteopontin, GAPDH (Santa Cruz Biotechnology, Santa Cruz, CA, USA) or β-tubulin (Abcam, Eugene, OR, USA). After washing the membrane, it was incubated with secondary horseradish peroxidase-conjugated antibodies for an hour. Signals were detected with enhanced chemiluminescence (GE Healthcare, Tokyo, Japan) and scanned with the image analyzer LAS-4000 (Fuji Film, Tokyo, Japan). Band intensities were determined using the ImageJ software [13].

### RNA extraction, cDNA synthesis and human MAPK signaling targets PCR array

Approximately 1.0 x 10^5^ cells per well were plated into a 6-well plate and, 12 hours later, were treated with or without 10 μM sorafenib (Cayman Chemical, Ann Arbor, MI, USA) [14]. Cellular RNA was extracted by an RNeasy Mini Kit (Qiagen, Hilden, Germany). cDNA was synthesized with an RT^2^ First Strand cDNA Kit (Qiagen) according to the manufacturer's protocol [15]. A human MAPK signaling pathway PCR array was purchased from Qiagen. A real-time PCR array based on the SYBR Green method was performed onto a 7300 Real-Time PCR system (Applied Biosystems, Foster, CA, USA). The cycling program was as follows: 95°C for 10 minutes for 1 cycle, then 40 cycles of 95°C for 15 seconds and 60°C for 1 minute. The house-keeping genes beta-2-microglobulin (B2M), hypoxanthine phosphoribosyltransferase 1 (HPRT1), ribosomal protein L13a (RPL13A), glyceraldehyde-3-phosphate dehydrogenase (GAPDH) and actin beta (ACTB) served as internal control. Data were analyzed using the RT^2^ Profiler PCR Array Data Analysis software (http://pcrdataanalysis.sabiosciences.com/pcr/arrayanalysis.php).

### Transfection of siRNA

The siRNA against c-Jun (si-c-Jun) and control siRNA (si-C) were purchased from Santa Cruz Biotechnology [11]. Transfections were performed with 50 nM si-c-Jun, or 50 nM si-C using Effectene Transfection Reagents (Qiagen) according to the manufacturer’s protocol [15].

### Overexpression of MEKK and reporter assay for AP-1 activation

To overexpress c-Jun, MEKK upstream of c-Jun was overexpressed using the plasmid pMEKK (Agilent Technologies, Tokyo, Japan) [11]. The combination of c-Jun with c-Fos forms the activator protein-1 (AP-1) early response transcription factor. Cells were seeded onto a 6-well plate. After 24 hours, 0.2 μg of the reporter plasmid pAP-1-luc (PathDetect Cis-Reporting Systems; Agilent Technologies, Santa Clara, CA, USA) and 0.01 μg pMEKK were co-transfected using Effectene transfection reagents (Qiagen). After incubation for 48 hours, the cells were harvested using reporter lysis buffer (Toyo Ink, Tokyo, Japan), and the luciferase activities were determined by a Picagene system (Toyo Ink) using a luminometer (Luminescencer-JNR II AB-2300, ATTO).

### MTS assay

To determine cell proliferation, a CellTiter 96 AQueous One Solution Cell Proliferation Assay (Promega, Madison, WI, USA) was performed. Living cells converted 5-(3-carboxymethoxyphenyl)-2-(4,5-dimenthylthiazoly)-3-(4-sulfophenyl) tetrazolium, inner salt (the MTS tetrazolium compound) to formazan. The cells were grown in 96-well plates for 24 hours before the medium was replaced with 0.2 mL of fresh medium containing sorafenib. After incubating the cells for 12 hours, 20 μL of MTS solution was added to each well. Four hours later, the absorbance at 490 nm of each well was measured with the iMark Microplate Absorbance Reader (Bio-Rad).

### Apoptosis assay

Quantification of apoptosis was performed with the APOPercentage apoptosis assay (Biocolor, Belfast, Northern Ireland). Purple-red stained cells were identified as apoptotic cells by light microscopy. Purple-red cells/fields of 400-fold views were counted as previously described [11].

### Caspase-3/-7 activity

Determination of caspase-3 and -7 activity was performed with a Caspase-Glo 3/7 assay (Promega) according to the manufacturer’s instructions [11]. Luminescence was measured using Luminescencer-JNR II AB-2300 (ATTO).

### Statistical analysis

Data are expressed as mean ± standard deviation (SD). Comparisons were analyzed using Student’s t test. Significance was defined as a P-value lower than 0.05.

## Results

### Human hepatoma cell lines possessed sorafenib resistance after treatment with 10 μM sorafenib for 12 hours

**Fig 1:**
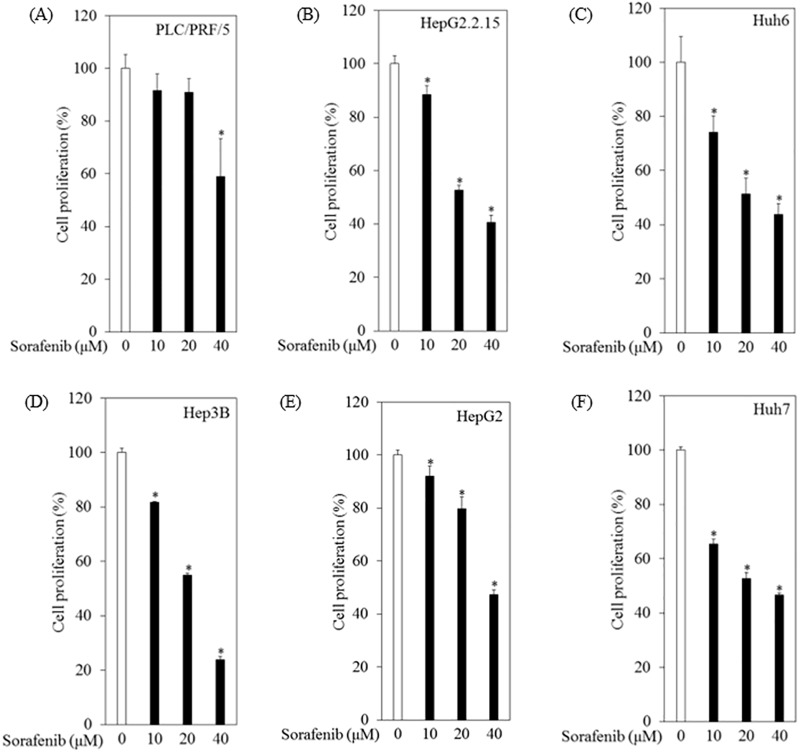
Effects of sorafenib on cell proliferation in human hepatoma cell lines. (A) PLC/PRF/5, (B) HepG2.2.15, (C) Huh6, (D) Hep3B, (E) HepG2 and (F) Huh7 cells. The cells were treated with sorafenib at the indicated concentrations for 12 hours, and cell proliferation was evaluated by MTS assay (Promega). Data are presented as mean ± SD of triplicate samples. *p < 0.*05* compared to the untreated control.

To explore the mechanism underlying sorafenib resistance, we first examined the effects of sorafenib on cell proliferation in 6 human hepatoma cell lines: PLC/PRF/5, HepG2.2.15, Huh6, Hep3B, HepG2 and Huh7 (Fig 1). Cells were incubated with sorafenib at various concentrations for 12 hours, and cell proliferation was evaluated by an MTS Cell Proliferation assay. Although sorafenib reduced cell proliferation in a dose-dependent manner, we noticed that when the cells were incubated with 10 μM sorafenib, only 8.4%, 11.6%, 25.8%, 18.2%, 7.9% and 34.5% inhibition was observed in PLC/PRF/5, HepG2.2.15, Huh6, Hep3B, HepG2 and Huh7 cells, respectively, compared to untreated controls (Fig 1). Treatment with 20 μM sorafenib for 12 hours significantly reduced cell proliferation in all cell lines except PLC/PRF/5 cells, compared to untreated controls (Fig 1). Treatment with 40 μM sorafenib for 12 hours significantly reduced cell proliferation in all cell lines compared to untreated controls (Fig 1). When cells were incubated with sorafenib at 10 μM for 24 hours, 51.6%, 36.5%, 16%, 40.2%, 10.8% and 29.1% inhibition was observed in PLC/PRF/5, HepG2.2.15, Huh6, Hep3B, HepG2 and Huh7 cells, respectively, compared to untreated controls. The highest achievable clinical blood concentration of sorafenib is 10 μM [14]. In total, 65.5% - 92.1% of human hepatoma cell lines were viable in the treatment with sorafenib at this concentration for 12 hours.

### c-Jun was upregulated after treatment with sorafenib in human hepatoma cell lines

**Fig 2:**
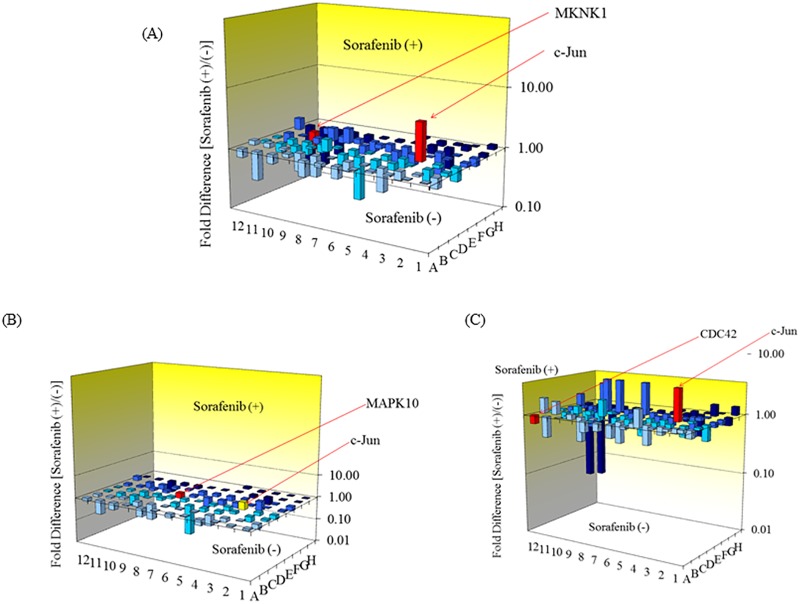
Changes of MAPK-signaling pathway-associated genes in human hepatoma cell lines treated with or without sorafenib. Six human hepatoma cell lines were treated with or without 10 μM sorafenib for 12 hours. (A) Total expression of 6 human hepatoma cells (PLC/PRF/5, HepG2.2.15, Huh6, Hep3B, HepG2 and Huh7). Expression of 2 genes significantly changed after 12 hours of treatment with sorafenib: Jun proto-oncogene (c-Jun) and MAP kinase interacting serine/threonine kinase 1 (MKNK1), which are shown in red. (B) Human hepatoma cells without HBV genome integration (Huh6, HepG2 and Huh7). Mitogen-activated protein kinase 10 (MAPK10) expression significantly changed after 12 hours of treatment with sorafenib. MAPK10 is shown in red, and c-Jun is shown in yellow. (C) Human hepatoma cells with HBV integration (PLC/PRF/5, HepG2.2.15 and Hep3B). Expression of 2 genes significantly changed after 12 hours of treatment of sorafenib: c-Jun and cell division cycle 42 (GTP binding protein, 25 kDa) (CDC42), which are shown in red.

Sorafenib is a multiple kinase inhibitor that inhibits Raf/MEK/MAPK signaling. We expected that some genes in this signal pathway might be overexpressed in sorafenib-resistant cells. Next, we examined the signaling pathway related to 84 MAPK-signaling pathway-associated genes in 6 human hepatoma cell lines treated with or without sorafenib (Fig 2, S1 Fig and Tables A-G in S1 File). Among these genes, MAP kinase interacting serine/threonine kinase 1 (MKNK1) was significantly downregulated (0.49-fold, *p = 0*.*0128*) in human hepatoma cells treated with 10 μM sorafenib (Fig 2A). These results also indicated that sorafenib could inhibit MAPK-signaling pathway-associated gene expression. Among those genes, we found that c-Jun was the only gene significantly upregulated (4.27-fold, *p = 0*.*0125*) among a total of 6 cell lines treated with 10 μM sorafenib.

We compared gene expression in human hepatoma cells without HBV genome integration (Huh6, HepG2 and Huh7) treated with or without 10 μM sorafenib (Fig 2B). Mitogen-activated protein kinase 10 (MAPK10), which is known as JNK3, was significantly downregulated (0.58-fold, *p = 0*.*0206*) among the cells treated with 10 μM sorafenib. MKNK1 tended to be downregulated (0.56-fold, *p = 0*.*0890*) in cells treated with 10 μM sorafenib.

We also compared gene expression in human hepatoma cells with HBV genome integration (PLC/PRF/5, HepG2.2.15 and Hep3B) treated with or without 10 μM sorafenib (Fig 2C). c-Jun was significantly upregulated (8.58-fold, *p = 0*.*000935*) and cell division cycle 42 (CDC42: GTP binding protein, 25 kDa) was significantly downregulated (0.72-fold, p = 0.0389) in cells treated with 10 μM sorafenib.

### Phosphorylation of c-Jun increased after treatment with sorafenib in human hepatoma cell lines

**Fig 3:**
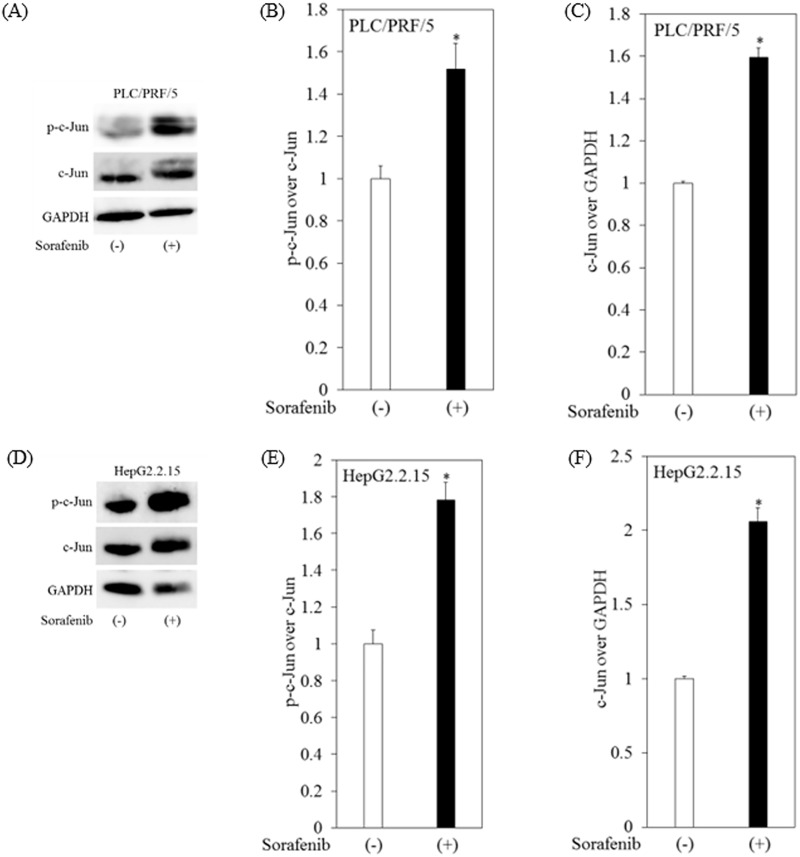
Sorafenib enhances expression and phosphorylation of c-Jun in human hepatoma cell lines. (A)-(C) Western blot analyses of phosphorylated-c-Jun (p-c-Jun), c-Jun and GAPDH expression in PLC/PRF/5 cells treated with or without 10 μM sorafenib for 12 hours. (D)-(F) Western blot analyses of p-c-Jun, c-Jun and GAPDH expression in HepG2.2.15 cells treated with or without 10 μM sorafenib for 12 hours. (B, C, E, F) Densitometric analyses were performed using ImageJ software. Data are presented as mean ± SD of triplicate samples. *p < 0.*05* compared to untreated control.

Compared to untreated cells, c-Jun gene expression was 17.29-, 12.94-, 8.62-, 2.82-, 1.17-, and 0.95-fold in 10 μM sorafenib-treated PLC/PRF/5, HepG2.2.15, Huh6, Hep3B, HepG2, and Huh7 cells, respectively. So we mainly used PLC/PRF/5 and HepG2.2.15 for additional analyses. We then examined the effects of sorafenib in the phosphorylation of c-Jun and c-Jun protein expression in PLC/PRF/5 and HepG2.2.15 cells treated with 10 μM sorafenib. Treatment of PLC/PRF/5 cells with sorafenib was associated with 1.51-fold and 1.59-fold increases in the phosphorylation of c-Jun and c-Jun protein expression, respectively (Fig 3A–3C), and treatment of HepG2.2.15 cells with sorafenib was associated with 1.78- and 2.05-fold increases in the phosphorylation of c-Jun and c-Jun protein expression, respectively (Fig 3D–3F). These results suggested the possibility that the expression and phosphorylation of c-Jun could be associated with sorafenib resistance.

### Knockdown of c-Jun enhanced sorafenib-induced apoptosis in human hepatoma cells

**Fig 4:**
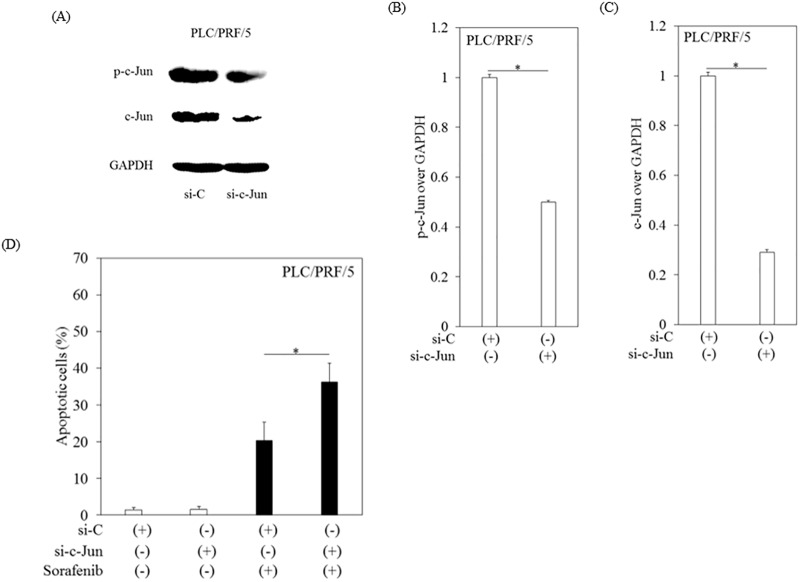
Knockdown of c-Jun enhanced sorafenib-induced apoptosis in human hepatoma PLC/PRF/5 cells. (A)-(C) Validation of siRNAs si-c-Jun and si-control (si-C). Lysates from transfected cells were immunoblotted with antibodies against p-c-Jun, c-Jun or GAPDH. GAPDH was used as internal control. Densitometric analyses were performed with ImageJ software. Data are presented as mean ± SD of triplicate samples. (D) Apoptosis in PLC/PRF/5 cells treated with or without 7.5 μM sorafenib for 48 hours after transfection with each siRNA. Apoptosis was determined by an APOPercentage apoptosis assay (Biocolor). (B, D) Densitometric analyses were performed with ImageJ software. Data are presented as mean ± SD of triplicate samples. *p < 0.*05* between two groups.

After the efficacy of siRNAs was confirmed in hepatocytes (Fig 4A–4C), apoptotic cell death in PLC/RPF/5 cells treated with or without sorafenib following transfection with either si-c-Jun or si-C was analyzed using an APOPercentage apoptosis assay (Fig 4D). We treated the cells with 7.5 μM sorafenib for 48 hours. We used this condition because almost all cells were apoptotic when both siRNAs-transfected cells were treated with 10 sorafenib for 12 hours. In sorafenib-treated PLC/RPF/5 cells transfected with si-c-Jun, apoptotic cells were significantly increased compared to sorafenib-treated PLC/RPF/5 cells transfected with si-C. Compared to sorafenib-treated and si-C-transfected control Hep3B, HepG2 and Huh7 cells, significant increases in apoptotic cells were also observed in the same cell lines when sorafenib-treated and si-c-Jun-transfected (1.81-, 1.75- and 1.47-fold increase, respectively; *p < 0*.*05* compared to si-C-transfected control cells).

### Overexpression of c-Jun by transfection of pMEKK into PLC/RPF/5 cells impaired sorafenib-induced apoptosis in PLC/RPF/5 cells

**Fig 5:**
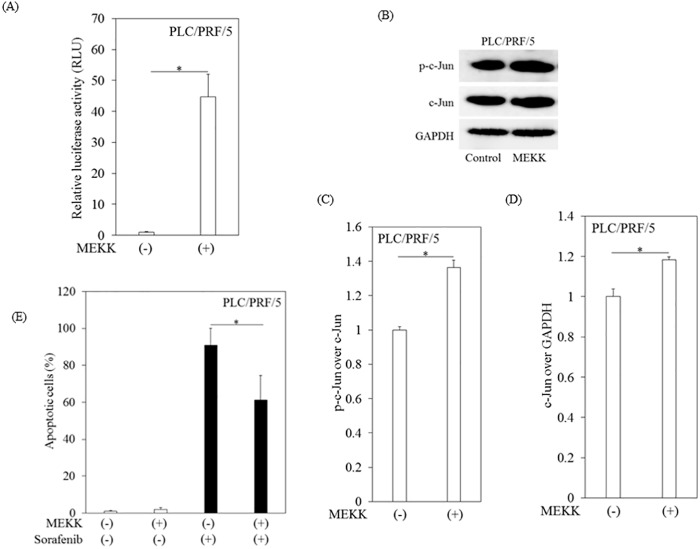
Overexpression of c-Jun by transfection of pMEKK impaired sorafenib-induced apoptosis in human hepatoma PLC/PRF/5 cells. (A) AP-1 activation following the transfection of pMEKK into PLC/PRF/5 cells. (B)-(D) Phosphorylated-c-Jun (p-c-Jun) and expression of c-Jun protein were enhanced by transfection of pMEKK into PLC/RPF/5 cells. Densitometric analyses were performed with ImageJ software. (E) Apoptosis in PLC/PRF/5 cells treated with or without 10 μM sorafenib for 12 hours after transfection of pMEKK or control vectors. The number of apoptotic cells was determined by APOPercentage apoptosis assay (Biocolor). Data are presented as the mean ± SD of triplicate samples. *p < 0.*05* between groups.

We investigated the effects of AP-1 activation on sorafenib-induced apoptosis. As shown in Fig 5A, transfection of the pMEKK plasmids into PLC/RPF/5 cells enhanced AP-1 activity in a reporter assay. In PLC/RPF/5 cells transfected with pMEKK, the expression and phosphorylation of c-Jun increased (Fig 5B–5D). Transfection with pMEKK vectors significantly reduced apoptosis in PLC/RPF/5 cells treated with sorafenib (Fig 5E). Overall, these results indicate that c-Jun is one of the factors responsible for sorafenib resistance in human hepatoma cells.

### JNK inhibitor SP600125 enhanced sorafenib-induced apoptosis in human hepatoma cells

**Fig 6:**
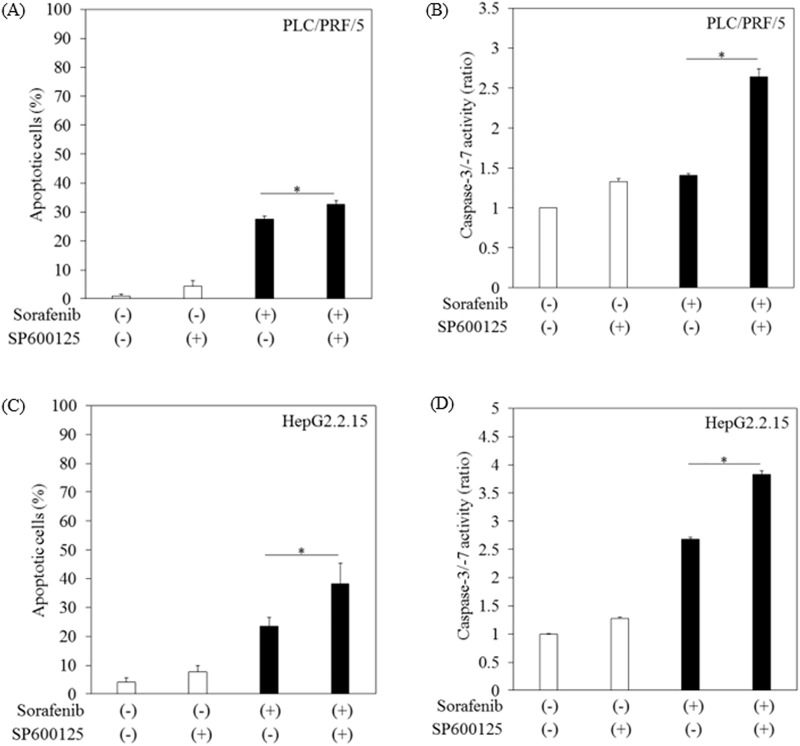
SP600125 enhanced sorafenib-induced apoptosis in human hepatoma cell lines. (A, B) PLC/RPF/5, (C, D) HepG2.2.15. Apoptosis in cells treated with or without 10 μM sorafenib for 12 hours after treatment with or without 45 μM SP600125 for 12 hours. (A, C) The number of apoptotic cells was determined by APOPercentage apoptosis assay (Biocolor). (B, D) Caspase-3/-7 activity was measured by Caspase-Glo 3/7 assay (Promega). Data are presented as mean ± SD of triplicate samples. *p < 0.*05* between two groups.

SP600125 prevented the activation of JNK. We next examined the effects of SP600125 on sorafenib-induced apoptosis in human hepatoma cells (Fig 6). Apoptosis was analyzed in PLC/RPF/5 cells treated with or without 10 μM sorafenib for 12 hours after treatment with or without 45 μM SP600125 for 12 hours. We observed a significantly higher proportion of apoptotic cells with the combination of sorafenib and SP600125 in the APOPercentage assay (Fig 6A). Activation of caspase-3/-7 also supported these results (Fig 6B). In HepG2.2.15 cells, the results were similar to those obtained for PLC/RPF/5 cells (Fig 6C and 6D). In addition, we also observed a significantly higher proportion of apoptotic cells with the combination of sorafenib and SP600125 in HepG2 cells by APOPercentage assay (5.58-fold; *p < 0*.*05* compared to sorafenib-treated control cells).

We also examined the effects of SP600125 on the anti-cancer-drug-induced apoptosis in PLC/RPF/5 cells. The degree of apoptosis was similar in the presence of 16 μM cis-diamminedichloro-platinum (CDDP) with or without 45 μM SP600125 (11.2 ± 4.4% vs. 6.8 ± 0.69%, respectively). Apoptosis was also similar in the presence of 0.5 μg/mL 5-fluorouracil (5FU) with or without 45 μM SP600125 (7.7 ± 0.7% vs. 6.0 ± 2.0%, respectively). However, apoptosis in the presence of 100 nM gemcitabine (GEM) with 45 μM SP600125 was higher than that in the absence of GEM (2.7% ± 0.5% vs. 6.7 ± 1.6%, *p = 0*.*040*).

### Sorafenib enhanced expression of osteopontin, an AP-1 target gene, in human hepatoma cell lines

**Fig 7:**
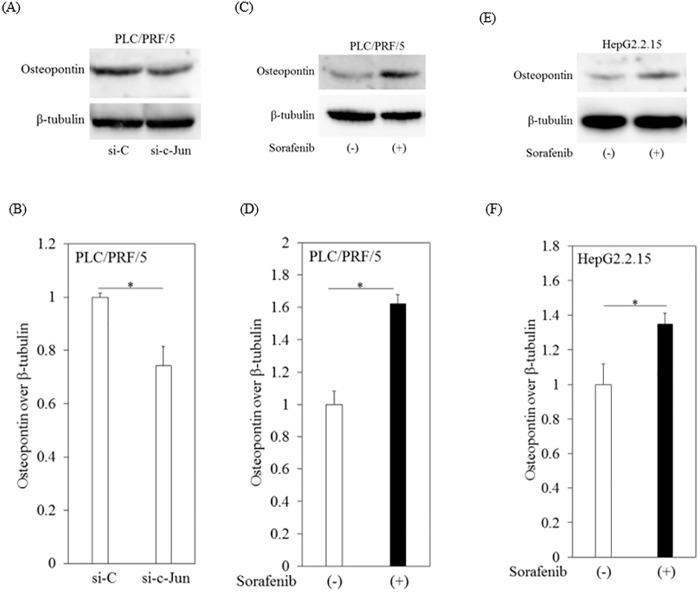
Sorafenib enhanced expression of osteopontin, an AP-1 target gene, in human hepatoma cell lines. (A, B) Knockdown of c-Jun decreased expression of osteopontin after 48 hours of transfection into PLC/PRF/5 cells with siRNA against c-Jun (si-c-Jun) or si-control (si-C). Lysates from transfected cells were immunoblotted with antibodies against osteopontin or β-tubulin. β-tubulin was used as internal control. (C, D) Western blot analyses of osteopontin and β-tubulin expression in PLC/PRF/5 cells treated with or without 10 μM sorafenib for 12 hours. (E, F) Western blot analyses of osteopontin and β-tubulin expression in HepG2.2.15 cells treated with or without 10 μM sorafenib for 12 hours. Densitometric analyses were performed with ImageJ software. Data are presented as mean ± SD of triplicate samples. *p < 0.*05* between two groups.

To investigate the mechanism further, we focused on osteopontin, an established AP-1 target gene [16]. We confirmed that knockdown of c-Jun led to a decrease in the expression of osteopontin in PLC/RPF/5 cells (Fig 7A and 7B). After treatment with sorafenib in human hepatoma cell lines, we also observed that the expression of osteopontin increased (Fig 7C–7F).

## Discussion

In this study, we focused on the transcription factor c-Jun and demonstrated that c-Jun was involved in the resistance of sorafenib in certain human hepatoma cell lines. We showed that c-Jun and its phosphorylation determined sorafenib-induced apoptosis in human hepatoma cell lines. Inhibiting c-Jun could enhance the apoptosis of human hepatoma cells in the presence of sorafenib. We also demonstrated that osteopontin may contribute to these phenomena. Our observations indicated that c-Jun plays an important role in sorafenib resistance in HCC.

The RAS-RAF-MEK-MAPK pathway is a key signal transduction pathway in cells and is constitutively active in HCCs [17]. It is also responsible for poor prognosis and drug resistance [7]. Targeting the responsible proteins, such as the hepatocyte growth factor (HGF) receptor and the phosphatidylinositol-4,5-bisphosphate 3-kinase (PI3K)/ AKT serine/threonine kinase (AKT) pathways, are also essential [7].

Drug resistance was also associated with epithelial-mesenchymal transition (EMT) in HCC [18]. In anoikis-resistant HCC cells, which are highly sorafenib-resistant and induce EMT, cellular apoptosis was associated with c-Jun [19]. OCT4, one of the pluripotency genes, regulates EMT and is associated with chemoresistance [20]. Positive feedback regulation of OCT4 and c-Jun could expedite cancer stemness in liver cancer [21].

Fibrosis, which is one of the features of collagen-rich microenvironments, could reduce the efficacy of sorafenib by impairing delivery of chemotherapeutics and promoting aggressive neoplastic cell behavior [22–24]. JNK is an important component that converts external stimuli into a wide range of cellular responses, such as fibrosis [23].

Sorafenib is a bi-aryl urea: N-(2-trifluoromethyl-4-chlorophenyl)-N´-(4-[2-methylcarbamoyl pyridine-4-yl] oxyphenyl) urea [14]. Sorafenib is bound to human plasma proteins, and albuminemia influences the total clearance of sorafenib [25]. Albumin is synthesized in the liver and in cirrhotic liver, and this mostly occurs in the background in livers of HCC patients, and seems to affect the blood concentration of sorafenib. Sorafenib is metabolized by CYP3A4 and uridine diphosphate glucuronosyltransferase 1A9 (UGT1A9) in the liver [26]. Co-administration of a proton pump inhibitor led to a significant drop in sorafenib exposure [27]. Clinicians should also pay attention to these factors.

Trierweiler et al. [16] reported that c-Jun/AP-1 promoted HBV-related liver tumorigenesis in mice. Cheng et al. [28] demonstrated that sorafenib-treated patients with HBV-associated HCC had fewer survival benefits than in those with non-HBV related HCC. Half maximal (50%) inhibitory concentration (IC50) for sorafenib was significantly higher in HBV-positive HCC cells than in those without HBV infection [29]. We also observed that c-Jun was highly upregulated in HBV-associated human hepatoma cells treated with sorafenib (Fig 2B and 2C).

We also compared gene expression in the human hepatoma cell line Huh7 harboring HCV subgenomic replicon [30] treated with or without 10 μM sorafenib. However, c-Jun was not significantly upregulated. The HCV-associated liver cancer cell lines do not include HCV genome integration or full-length HCV RNA [30]. Further study will be needed to investigate virally mediated oncogenesis.

In HCC-patients treated with sorafenib, the expression of phosphorylated c-Jun in HCC was significantly higher in the non-responder group than in the responder group [31]. Chen et al. [32] also reported that activation of c-Jun predicted a poor response to sorafenib in HCC. Our results supported these facts. Phosphorylated-JNK was correlated with the activation of c-Jun/AP-1 proteins in HCC [33].

CD133, identified as one of the cancer stem cell markers, contributed to the initiation and growth of HCC [31]. Phosphorylated c-Jun was also correlated with CD133 in HCC [31]. It was reported that a high percentage of cells was arrested in the G2 phase 48 hours after treatment with a JNK inhibitor [34]. Combination treatment with SP600125 and TNF-related apoptosis-inducing ligand (TRAIL) led to apoptosis in human hepatoma cells [34]. SP600125 is known to inhibit other genes such as TNF, which is one of the nuclear factor-kappa B (NF-κB) target genes [35]. Expression of conserved helix-loop-helix ubiquitous kinase (CHUK), an inhibitor of the transcription factor NF-κB complex, was unaltered either with or without sorafenib treatment (Table F in S1 File).

Osteopontin is a multi-functional cytokine that is involved in cell survival, migration and chemotherapy-resistance, including sorafenib-resistance in patients with metastatic renal cell carcinoma [36, 37]. We also observed that sorafenib upregulated osteopontin expression in human hepatoma cell lines and that c-Jun played a role to some extent in this step (Fig 7). Further study of these trends will be needed.

Although there are conflicting opinions about whether sorafenib also suppresses JNK-dependent apoptosis [38, 39], c-Jun/AP-1 is one of the more attractive targets for the chemotherapy of cancers, including HCC [40]. In conclusion, c-Jun was associated with sorafenib resistance in human hepatoma cell lines. Modulation of c-Jun and phosphorylated c-Jun might be a potential tool for improving the response to sorafenib in HCC patients.

## Supporting information

S1 Fig Heat map analysis for the expression of MAPK-signaling pathway-associated genes in 6 human hepatoma cells (PLC/PRF/5, HepG2.2.15, Huh6, Hep3B, HepG2 and Huh7) treated with or without sorafenib. The six human hepatoma cell lines were treated with or without 10 μM sorafenib for 12 hours. Red color indicates genes expressed higher in cells treated with sorafenib than in those without sorafenib. Green color indicates genes expressed lower in cells treated with sorafenib than in those without sorafenib. Arrows indicate Jun proto-oncogene (JUN) and conserved helix-loop-helix ubiquitous kinase (CHUK).
(TIF)

S1 File Results of human MAPK signaling targets PCR array in this study. Table A. List of analyzed genes.
Table B. Gene expression profiles in 6 human hepatoma cells treated with sorafenib.
Table C. Gene expression profiles in 6 human hepatoma cells treated without sorafenib.
Table D. List of housekeeping genes.
Table E. Overview of the PCR Array Performance and quality control.
Table F. Results of PCR arrays.
Table G. Calculation.
(XLS)
