# Supplementary figures and images for: Overexpression of c-Jun contributes to sorafenib resistance in human hepatoma cell lines
Source: PLoS One. 2017 Mar 21;12(3):e0174153. doi: 10.1371/journal.pone.0174153 (PMC5360329; doi:10.1371/journal.pone.0174153)

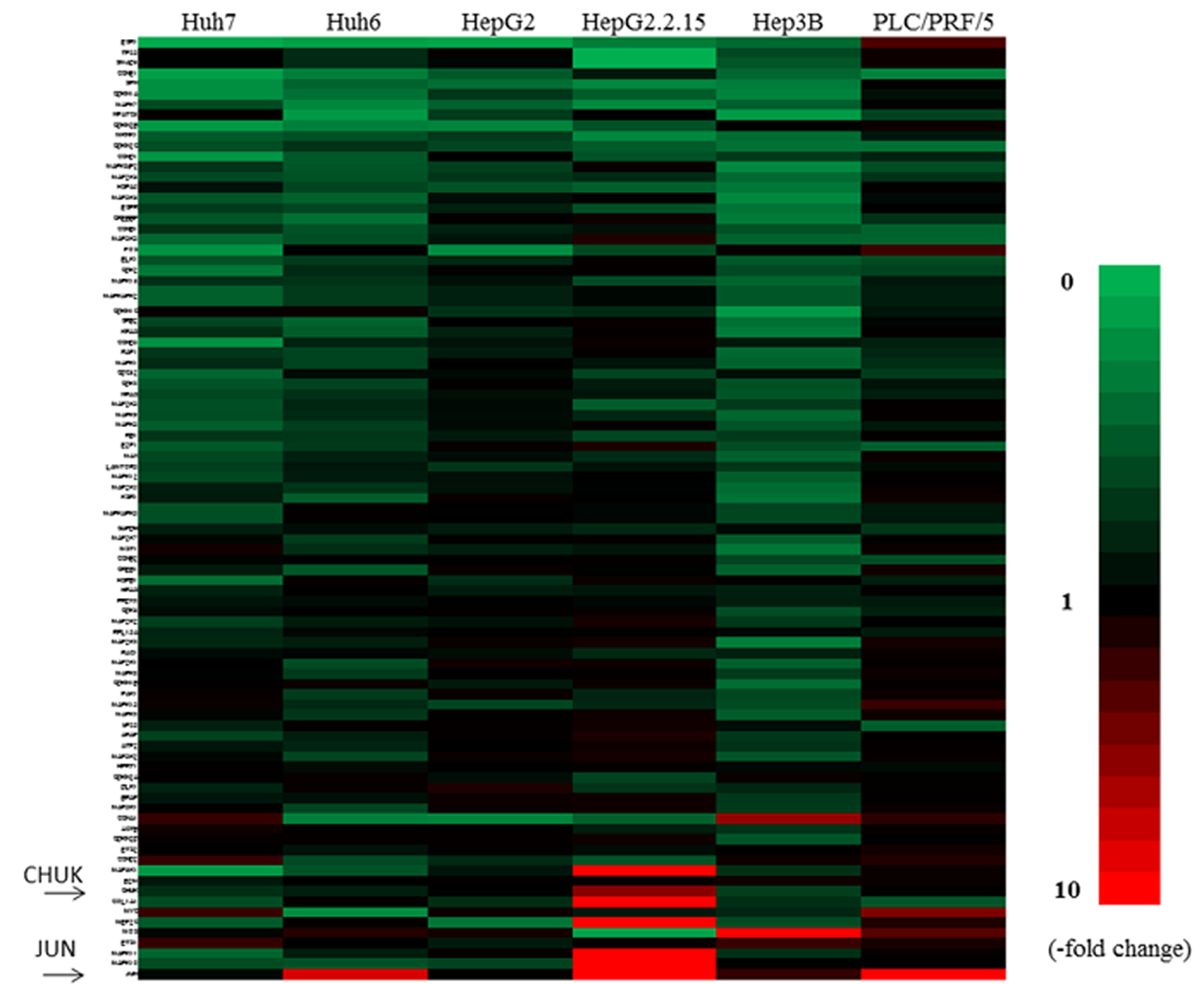

Supplement: S1 Fig — The six human hepatoma cell lines were treated with or without 10 μM sorafenib for 12 hours. Red color indicates genes expressed higher in cells treated with sorafenib than in those without sorafenib. Green color indicates genes expressed lower in cells treated with sorafenib than in those without sorafenib. Arrows indicate Jun proto-oncogene (JUN) and conserved helix-loop-helix ubiquitous kinase (CHUK). (TIF) [file pone.0174153.s001.tif]
